# Pili canaliculi as manifestation of giant axonal neuropathy*

**DOI:** 10.1590/abd1806-4841.20164677

**Published:** 2016

**Authors:** Hiram Larangeira de Almeida Jr., Gilberto Garcias, Ricardo Marques e Silva, Stela Laner Batista, Fernanda Pasetto

**Affiliations:** 1 Universidade Federal de Pelotas (UFPel) – Pelotas (RS), Brazil

**Keywords:** Giant axonal neuropathy, Hair, Hair diseases, Microscopy, electron, scanning

## Abstract

Giant axonal neuropathy is a rare autosomal recessive neurodegenerative disease. The condition is characterized by neurons with abnormally large axons due to intracellular filament accumulation. The swollen axons affect both the peripheral and central nervous system. A 6-year old female patient had been referred to a geneticist reporting problems with walking and hypotonia. At the age of 10, she became wheelchair dependent. Scanning electron microscopy of a curly hair classified it as pili canaliculi. GAN gene sequencing demonstrated mutation c.1456G>A (p.GLU486LYS). At the age of 12, the patient died due to respiratory complications. Dermatologists should be aware of this entity since hair changes are considered suggestive of GAN.

## INTRODUCTION

Giant Axonal Neuropathy (GAN – OMIM # 256850) is a rare hereditary autosomal recessive neurodegenerative disease with unknown prevalence. *GAN* was originally *reported* in 1972 by *Berg* and colleagues. The condition is characterized by neurons with abnormally large axons due to intracellular filament accumulation. The swollen axons affect both the peripheral and central nervous system.^1,2^

GAN generally appears in infancy or early childhood, rarely at birth, and progresses to death. The onset of the disease usually presents with delay in the acquisition of abilities followed by gait disorders and progressive weakness in upper and lower limbs. It may also involve the brain nerves, resulting in facial weakness, optic atrophy and ophthalmoplegia. The disease evolves rapidly with the deterioration of the central nervous system showing epilepsy, cerebellar signs – ataxia, nystagmus and dysarthria – and signs of pyramidal tract damage, but rarely revealing mental disabilities. Another sign of the disease is dull, tightly-curled hair that is markedly different from the parents' in color and texture.^2^

Most individuals become wheelchair dependent in the second decade of life and eventually bedridden with severe polyneuropathy, ataxia and dementia, which may cause death in the third decade.

## CASE REPORT

A 10-year-old female patient consulted with a neurologist for the first time when she started having problems with walking at the age of 5 due to progressive loss of strength. At the age of 6, she was referred to a geneticist because of difficulty in ambulation caused by hypotonia in lower and upper limbs. Clinical examination revealed genu valgus and joint hypermobility. Karyotype, muscle enzymes and radiologic studies were normal.

At 7 the patient showed growth of pubic hair and a possible menstrual episode with normal LH, FSH, estrogen and progesterone levels. Magnetic resonance imaging of the brain revealed *pituitary cysts* and diffuse hypomyelination of the central nervous system.

**Figure 1:**
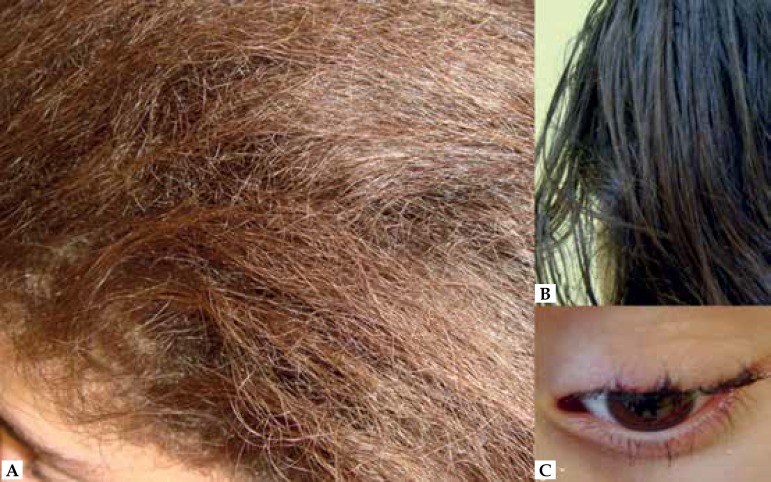
**A:** proband’s curly hair. **B:** mother’s normal hair for comparison. **C:** irregular eyelashes

At 10 she became wheelchair dependent. Orthopedic surgery was required to correct shortening of the Achilles tendons. Dermatological examination revealed curly hair different from her parents' (Figure 1A and 1B) with eyelash involvement (Figure 1C).

After GAN was suggested, we collected DNA from peripheral blood. Sequence analysis of GAN gene demonstrated the mutation c.1456G>A (p.GLU486LYS).

**Figure 2:**
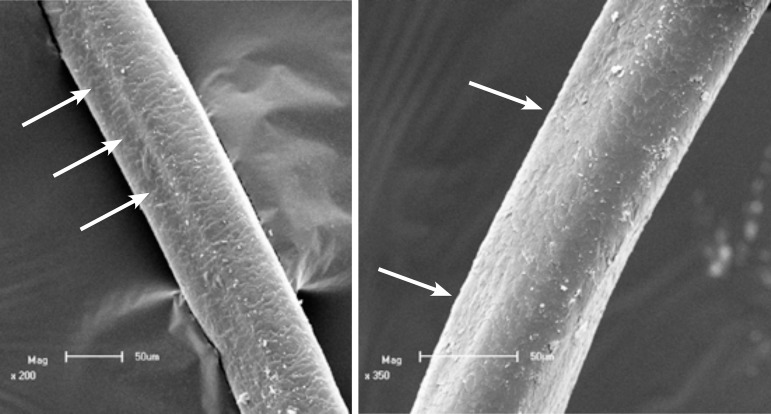
Scanning electron microscopy – low magnification showing longitudinal grooving (arrows) (x 200 and x 350).

**Figure 3:**
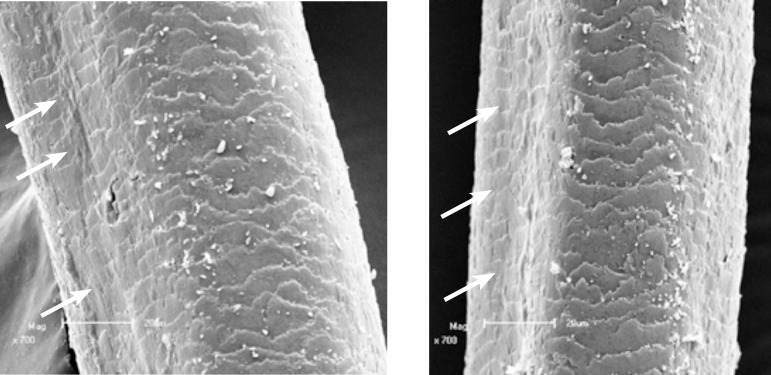
Scanning electron microscopy – high magnification showing grooving (arrows) and polygonal hair shape (x 700)

Scanning electron microscopy revealed longitudinal grooves on the surface of the hair at low magnification and a polygonal shape at higher magnification (Figures 2 and 3), typical findings of pili canaliculi.

The patient died at the age of 12 due to respiratory complications.

## DISCUSSION

Giant axonal neuropathy is caused by mutations in the GAN gene, which affect of the protein gigaxonin, leading to disorganization of neurofilaments (intermediate filaments of neuronal cells). The mutation may also lead to the axonal accumulation of proteins, hence the denomination giant axons.

One of the functions of the gigaxonin protein may be to maintain the architecture of other intermediate filaments such as keratins, which could explain one characteristic sign of the disease: the hair involvement.^5,6^ Some studies analyzed families affected by pili canaliculi – a cutaneous genetic manifestation without neurological involvement – in which patients presented uncombable hair or gradual hypotrichosis^3,4^. Ultrastructural hair examination in those patients revealed grooves on hair surface giving polygonal shapes (triangular, square, reniform) to the hair shaft.^4^ The results are similar to those presented by GAN patients' hair. Treiber-Held *et al.* reported cases of grooved hair in GAN patients, confirming our results.^2^

Since the results of *three-dimensional ultrastructural* hair analysis in our patient are suggestive of pili canaliculi, dermatologists should be aware of this entity because hair changes are considered suggestive of GAN.^1,7^
